# On a Magical Mystery Tour of Green Insecticide Research: Current Issues and Challenges

**DOI:** 10.3390/molecules25215014

**Published:** 2020-10-29

**Authors:** Giovanni Benelli

**Affiliations:** Department of Agriculture, Food and Environment, University of Pisa, via del Borghetto 80, 56124 Pisa, Italy; giovanni.benelli@unipi.it

**Keywords:** botanicals, beetles, essential oils, flies, mites, mosquitoes, insect pests, mode of action, nanoemulsions, nanoparticles, non-target toxicity, pesticide, ticks

## Abstract

The Editorial outlines recent research advances in green insecticide research. Particular attention is devoted to studies shedding light on the modes of action and non-target toxicity of natural substances of plant origin. Research focusing on the development of new formulations (including those relating to nano-objects) to magnify the effectiveness and stability of green insecticides in the field represents key advances. Herein, a carefully reviewed selection of cutting edge articles about green pesticide development recently published in *Molecules* is presented. The impact of sub-lethal doses of green insecticides on insect behavioral traits is still overlooked, representing a timely challenge for further research.

## 1. Introduction

As Academic Editor of *Molecules*, with research experience about insecticide development, I am delighted to provide this in-focus Editorial on the most exciting studies recently published by this journal. Managing arthropod pests and vectors is a key challenge nowadays, since the massive overuse of synthetic pesticides to manage their population leads to severe non-target effects on human health and the environment [1,2], coupled with the quick development of resistance in targeted species [3,4]. 

In this scenario, research focusing on new, effective and eco-friendly green insecticides and repellents is of the utmost importance, with special reference to the development of products for real-world use [5,6,7,8] characterized by multiple modes of action, which makes resistance development unlikely [9]. Therefore, *Molecules* strongly welcomes original research articles and reviews at the interface of phytochemistry and entomology, providing valuable “green” approaches for the management of arthropod pests and vectors. 

An impact factor higher than 3.5 (5-year value) and a first-decision time of just 12.3 days from manuscript submission, coupled with a rigorous peer-review process, highlights that this chemistry journal is performing excellently, being of high interest for a broad multidisciplinary readership, including pharmacologists, natural product researchers, entomologists and parasitologists, among others. 

*Molecules* is strongly interested in publishing research and reviews dedicated to green insecticide, acaricide and repellent development. Of note, authors and readers have access to several ongoing Special Issues focusing on various facets of this specific research area, thus achieving higher visibility if compared to regular issues. 

## 2. Editor’s Choice: A Selection of Cutting Edge Articles

Herein, I propose a selection of cutting edge articles about green insecticide development recently published in *Molecules* [9,10,11,12,13,14,15,16,17,18]. To my mind, these studies contribute to addressing timely research questions of both theoretical and applied importance. Overall, I hope that these papers will inspire future research on the topic, and I cordially invite the readers to consider submitting their green insecticide articles to *Molecules*.

Cutting edge articles about green insecticide research published on *Molecules*:

**Molecular Targets for Components of Essential Oils in the Insect Nervous System—A Review** by Milena Jankowska, Justyna Rogalska, Joanna Wyszkowska and Maria Stankiewicz *Molecules* 2018, 23(1), 34; https://doi.org/10.3390/molecules23010034


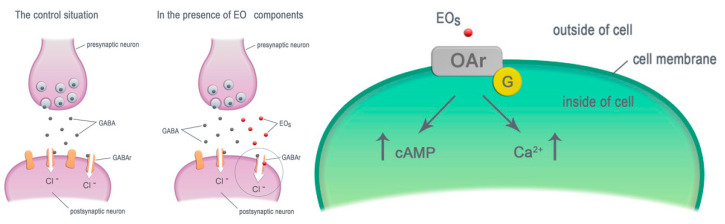


**Plant Natural Products for the Control of *Aedes aegypti*: The Main Vector of Important Arboviruses** by Maíra Rosato Silveiral Silvério, Laila Salmen Espindola, Norberto Peporine Lopes and Paulo Cézar Vieira *Molecules* 2020, 25(15), 3484; https://doi.org/10.3390/molecules25153484


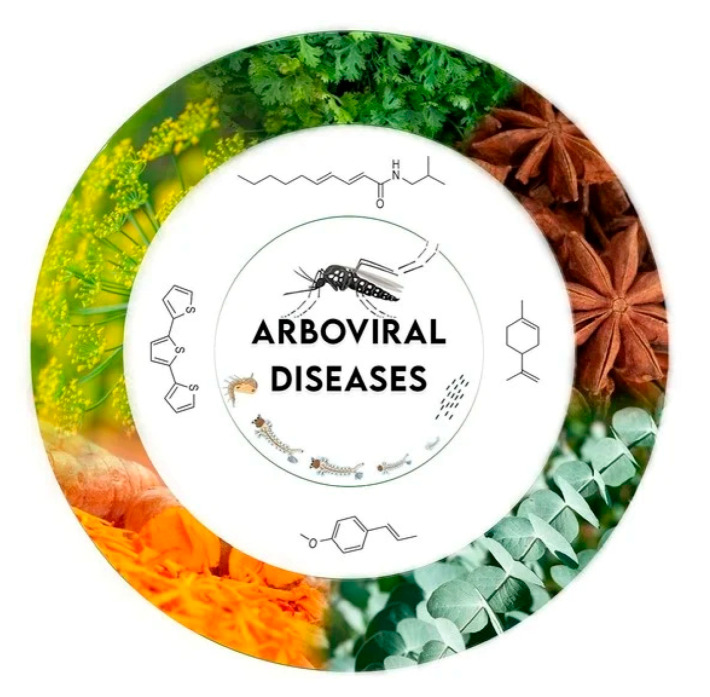


**Efficacy of *Origanum syriacum* Essential Oil against the Mosquito Vector *Culex quinquefasciatus* and the Gastrointestinal Parasite *Anisakis simplex*, with Insights on Acetylcholinesterase Inhibition** by Víctor López, Roman Pavela, Carlota Gómez-Rincón, Francisco Les, Fabrizio Bartolucci, Veronica Galiffa, Riccardo Petrelli, Loredana Cappellacci, Filippo Maggi, Angelo Canale, Domenico Otranto, Stefania Sut, Stefano Dall’Acqua and Giovanni Benelli *Molecules* 2019, 24(14), 2563; https://doi.org/10.3390/molecules24142563


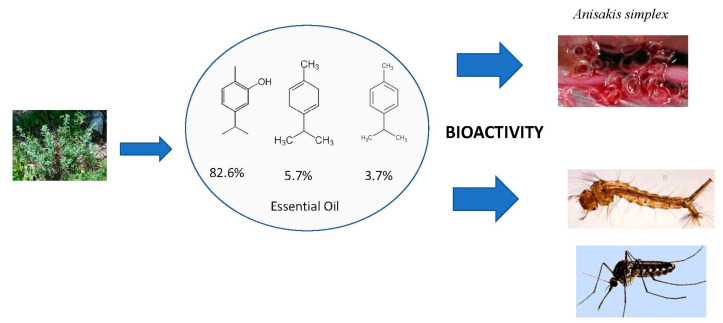


***Pimpinella anisum* Essential Oil Nanoemulsion Toxicity against *Tribolium castaneum*? Shedding Light on Its Interactions with Aspartate Aminotransferase and Alanine Aminotransferase by Molecular Docking** by Ahmed S. Hashem, Marwa, M. Ramadan, Amira A. A. Abdel-Hady, Stefania Sut, Filippo Maggi and Stefano Dall’Acqua *Molecules* 2020, 25(20), 4841; https://doi.org/10.3390/molecules25204841


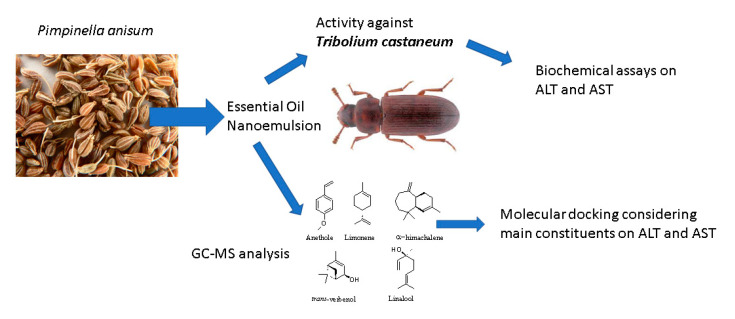


**Effect of Naringenin and Its Derivatives on the Probing Behavior of *Myzus persicae* (Sulz.)** by Katarzyna Stec, Joanna Kozłowska, Anna Wróblewska-Kurdyk, Bożena Kordan, Mirosław Anioł and Beata Gabryś *Molecules* 2020, 25(14), 3185; https://doi.org/10.3390/molecules25143185


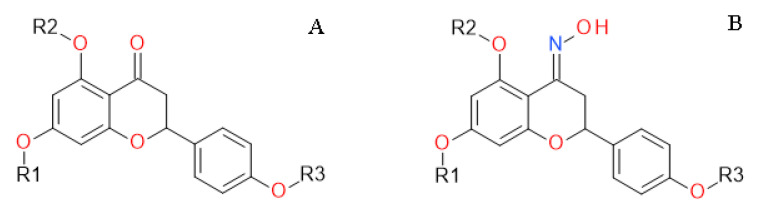


**Surfactantless Emulsions Containing Eugenol for Imidacloprid Solubilization: Physicochemical Characterization and Toxicity against Insecticide-Resistant *Cimex lectularius*** by Mariano Cáceres, Eduardo Guzmán, Agustín Alvarez-Costa, Francisco Ortega, Ramón G. Rubio, Carlos Coviella, Pablo L. Santo Orihuela, Claudia V. Vassena and Alejandro Lucia *Molecules* 2020, 25(10), 2290; https://doi.org/10.3390/molecules25102290


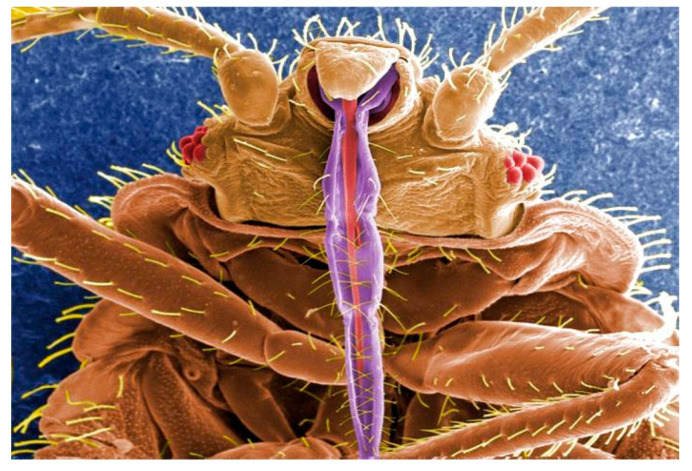


**Optimal Extraction of *Ocimum basilicum* Essential Oil by Association of Ultrasound and Hydrodistillation and Its Potential as a Biopesticide Against a Major Stored Grains Pest** by Eridiane da Silva Moura, Lêda Rita D’Antonino Faroni, Fernanda Fernandes Heleno, Alessandra Aparecida Zinato Rodrigues, Lucas Henrique Figueiredo Prates and Maria Eliana Lopes Ribeiro de Queiroz *Molecules* 2020, 25(12), 2781; https://doi.org/10.3390/molecules25122781


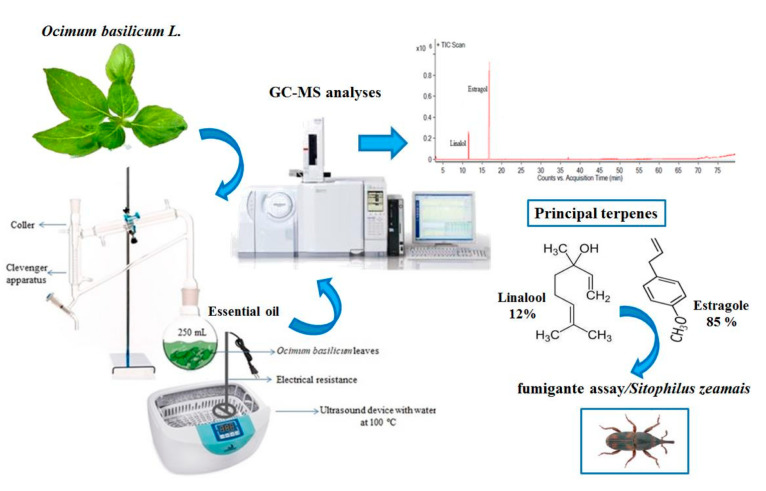


**Menthol Increases Bendiocarb Efficacy Through Activation of Octopamine Receptors and Protein Kinase A** by Milena Jankowska, Justyna Wiśniewska, Łukasz Fałtynowicz, Bruno Lapied and Maria Stankiewicz *Molecules* 2019, 24(20), 3775; https://doi.org/10.3390/molecules24203775


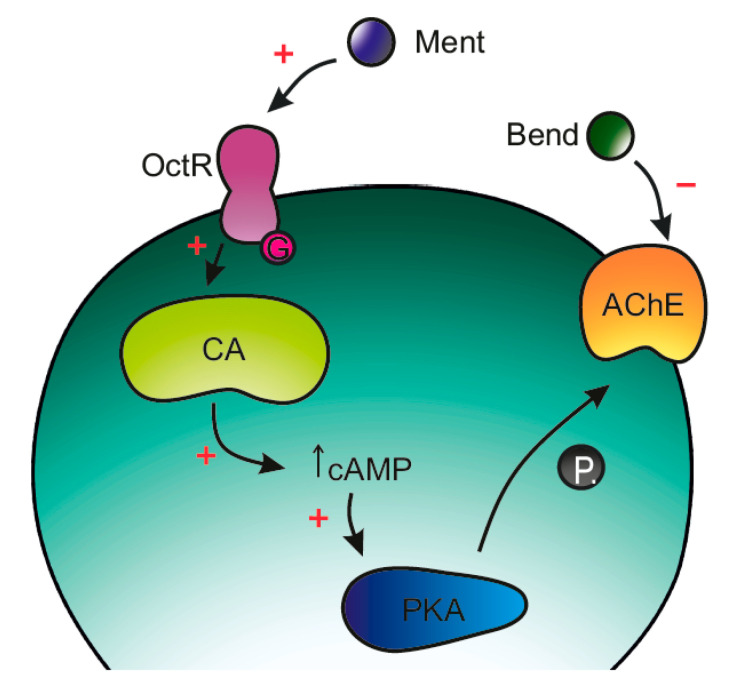


**Insights on the Larvicidal Mechanism of Action of Fractions and Compounds from Aerial Parts of *Helicteres velutina* K. Schum against *Aedes aegypti* L.** by Diégina A. Fernandes, Louise H. G. Oliveira, Hyago L. Rique, Maria de Fátima Vanderlei de Souza and Fabíola da Cruz Nunes *Molecules* 2020, 25(13), 3015; https://doi.org/10.3390/molecules25133015


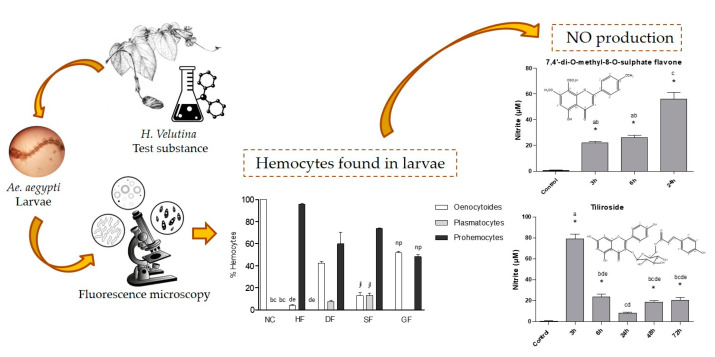


**Insecticidal Toxicities of Three Main Constituents Derived from *Trachyspermum ammi* (L.) Sprague ex Turrill Fruits against the Small Hive Beetles, *Aethina tumida* Murray** by Daniel Bisrat and Chuleui Jung *Molecules* 2020, 25(5), 1100; https://doi.org/10.3390/molecules25051100

## 3. Conclusions and Prospects

Overall, *Molecules* is strongly committed to publish green insecticide research. Peculiar attention is devoted to studies shedding light on the modes of action and non-target toxicity of natural substances of plant origin. Studies about the development of novel formulations (including nano-objects) to boost the effectiveness and stability of green insecticides under real-world conditions represent major advances in this research field. Of note, the impact of sub-lethal doses of green insecticides on insect behavioral traits is still overlooked, representing a timely and important challenge for further research.
